# Notes and News

**Published:** 1898-12-03

**Authors:** 


### Notes and News.

(Collected and Communicated.)

The annual meeting of the Surgical Aid Society will be held on Monday, December 5th, at the Cannon Street Hotel, at four p.m., the Lord Mayor in the chair.

According to the Maiyland *Medical Journal* an unexpected illustration of the way in which the dispensaries are abused in Baltimore occurred lately at the City Hospital Dispensary, when a man who had just applied for free treatment on the plea that he was unable to pay a physician dropped down dead, and in his pocket was found 1,500 dols. in notes.

It must be satisfactory to all supporters of progress and efficiency at Addenbrooke's Hospital, Cambridge, that the resolution put forward by the Committee, embodying alterations in the administration deemed necessary by them, has been confirmed by the majority of subscribers. At the poll, which was held on Monday, 145 voters were in favour of the resolution, against 94 dissentients.

In accordance with the plan set forth by the Prince of Wales, President of the Hospital Fund for London, last summer, a committee of gentlemen have visited and inspected carefully the various hospitals which applied for a share in this fund. The committee consisted of the following gentlemen: Sir Henry Burdett, Mr. Charles Burt, Sir Savile Crossley, Mr. J. G. Craggs, Dr. W. S. Church, Mr. John Croft, Viscount Duncannon, Mr. Frederick M. Fry, Mr. A. D. Fripp, Sir Trevor Lawrence, Mr. J. Pierpoint Morgan, jun., Mr. James C. Marshall, Mr. N. C. Macnamara, Dr. E. C. Perry, Mr. T. Pickering Pick, Mr. H. Power, Mr. Albert G. Sandeman, Sir Thomas Smith, Mr. Julius Wernher, and Mr. Alfred Willett. From these names it will be seen that nearly half may be termed experts in hospital management, and the various hospitals were visited by two or more visitors, in every instance one of these being a professional man. This is the first year in which any central funds, connected with the hospitals of London, have organised a system of personal visitation and inquiry, and the labours of the Distribution Committee will be greatly facilitated by the work thus done.

We are informed that, as the 20,000 copies of the "British Pharmacopœia," originally printed, have already passed into circulation, a reprint has been ordered, and the opportunity has been taken of indicating a few manifest errors and misprints. A slip containing these corrections may be obtained gratis from the publishers, Messrs. Spottiswoode and Co., Gracechurch Street, E.C.

We regret to learn that Mrs. Cope has resigned her appointment as secretary of the Royal Eye Hospital, Southwark, where for the past two and a half years she has done good and valuable work. Mrs. Cope is the fifth secretary of this hospital who has resigned during the past five years. The testimonials which Mrs. Cope has received from individual members of the council speak of her work in the highest terms, and great regret is felt at her departure.

"Truth" has just given a very bad account of the arrangements at the Port Hospital, Weymouth. It states that there are three wards for small-pox, scarlet fever, and typhoid respectively, which have been known to be all in requisition at the same time, whilst the whole staff for domestic and nursing purposes consists of a caretaker and his wife. The medical officer is the parish doctor. *Truth* depicts a scene at the hospital, when a visitor found the whole establishment asleep one evening, save a dying child who was in great pain and untended.

With a view to extending the use of the stamps issued on behalf of the Prince of Wales's Fund, it has been suggested that those hospitals which take payments from out-patients according to their means should adopt the stamps, giving them in exchange for the contributions received, so that each patient who does so shall have something to show that he is subscribing towards the cost of the benefits he is receiving. This idea has been experimentally adopted by one of the smaller hospitals, and already during the couple of months or so the plan has been in operation it has added materially to the income of that institution, while bringing the idea of regular subscription to the support of hospitals generally by means of the stamps to the notice not only of the patients, but of their friends and neighbours.

The following resolution respecting the retirement of Mr. Bland was passed at the last meeting of the committee of the Essex and Colchester Hospital: "That, in taking leave of Mr. Chas. E. Bland upon his retirement from the office of secretary to the Essex and Colchester Hospital, which he has held for the long term of nearly twenty-one years, the committee desire to record their sense of his uniform diligence in discharging the duties of his office, and particularly of his energy in organising for many years, over a large district, the Hospital Saturday Collections, and carrying out the occasional efforts for the improvement of the hospital (such as that made in connection with the recent Jubilee), the burden of which has fallen most heavily upon him, and for which he has received no extra remuneration. Further, in offering him their best wishes for his future success, they cannot fail to remember his unvarying courtesy in his long connection with the Hospital Committee."

